# Intervening on Calorie Intake or Eating Timing in Older Adults: Lessons Learned in the HALLO-P Trial

**DOI:** 10.1093/geroni/igaf122.1018

**Published:** 2025-12-31

**Authors:** Jason Fanning, Stephen Kritchevsky, Michael Miller, Denise Houston, Michael Walkup, Cynthia Stowe, W Jack Rejeski, Barbara Nicklas

**Affiliations:** Wake Forest University, Winston Salem, North Carolina, United States; Wake Forest University School of Medicine, Winston-Salem, North Carolina, United States; Wake Forest University School of Medicine, Winston-Salem, North Carolina, United States; Wake Forest University School of Medicine, Winston-Salem, North Carolina, United States; Wake Forest University School of Medicine, Winston-Salem, North Carolina, United States; Wake Forest University School of Medicine, Winston-Salem, North Carolina, United States; Wake Forest University, Winston-Salem, North Carolina, United States; Wake Forest University School of Medicine, Winston-Salem, North Carolina, United States

## Abstract

The Health, Aging and Later-Life Outcomes Pilot (HALLO-P) trial was designed to pilot in-person and remotely-delivered caloric restriction (CR and RCR) and time restricted eating (TRE) in preparation for a long-term clinical trial. Community-dwelling older adults with obesity or overweight with an indication for weight loss were randomized to 9 months of group-based CR, RCR, or TRE supported by self-monitoring technologies [i.e., a tablet application, activity monitor (all), wireless scale (CR and RCR)]. Intervention staff recorded attendance at sessions and noted intervention-related lessons learned as they arose, and participants provided feedback related to their experiences in the trial. Participants (N = 90; 67.19±4.91 years) attended 84.4% of sessions on average, engaged with study technologies 96.4% of days, and provided daily weights on > 4 days per week across the study (CR and RCR only). Participants reported high satisfaction with the program, with RCR participants being significantly more likely than TRE participants to report being satisfied with their program overall, intending to continue their dietary change post-intervention, and being willing to sustain their dietary approach and engage with study technologies over a 5-year period. Open-ended feedback underscored the value of the group-based structure of the program and remote delivery to reduce travel burden, the need for reduced burden associated with food logging, and the need to identify methods for enhancing perceived value of TRE among older adults. An intervention designed to promote CR, primarily delivered remotely and supported by self-monitoring technologies, is both feasible and acceptable to many older adults.

